# Carnauba Wax/Halloysite Nanotube with Improved Anti-Wetting and Permeability of Hydrophobic PVDF Membrane via DCMD

**DOI:** 10.3390/membranes11030228

**Published:** 2021-03-23

**Authors:** Wan Aisyah Fadilah Wae AbdulKadir, Abdul Latif Ahmad, Ooi Boon Seng

**Affiliations:** School of Chemical Engineering, Engineering Campus, Universiti Sains Malaysia, Nibong Tebal 14300, Penang, Malaysia; waecah992@gmail.com (W.A.F.W.A.); chobs@usm.my (O.B.S.)

**Keywords:** polymer-clay, natural wax, FOTS, anti-wetting, membrane distillation

## Abstract

The hydrophobic membranes have been widely explored to meet the membrane characteristics for the membrane distillation (MD) process. Inorganic metal oxide nanoparticles have been used to improve the membrane hydrophobicity, but limited studies have used nano clay particles. This study introduces halloysite nanotube (HNT) as an alternative material to synthesis a hydrophobic poly(vinylidene fluoride) (PVDF)-HNT membrane. The PVDF membranes were fabricated using functionalized HNTs (e.g., carnauba wax and 1H,1H,2H,2H-perfluorooctyl-trichlorosilane (FOTS)). The results were determined by Fourier transform infrared-attenuated total reflection, scanning electron microscope, goniometer and porometer to determine the desired hydrophobic membrane for direct contact membrane distillation (DCMD). The addition of FOTS-HNT (f_s_-HNT) and carnauba wax-HNT (f_w_-HNT) in the PVDF membrane enhanced the water contact angle (CA) to 127° and 137°, respectively. The presence of f_w_-HNT in the PVDF membrane exhibited higher liquid entry pressure (LEP) (2.64 bar) compared to f_s_-HNT in the membrane matrix (1.44 bar). The PVDF/f_w_-HNT membrane (Pf_w_-HNT) obtained the highest flux of 7.24 L/m^2^h with 99.9% salt removal. A stable permeability in the Pf_w_-HNT membrane was obtained throughout 16 h of DCMD. The incorporation of f_w_-HNT in the PVDF membrane had improved the anti-wetting properties and the membrane performance with the anti-fouling effect.

## 1. Introduction

Rising demand for freshwater production from saline or brackish natural water resources allowed for a broad utilization of available water resources by *Desalination* The high usage of seawater desalination for feed water application has overcome the water shortage issue. About 69% of water resources around the world has also implemented desalination plants for irrigation due to the cost-effective technology [1]. However, a challenge is raised for inland desalination when the disposal of brine (desalination by-products) requires a significant addition to the water production cost.

Removal of salt from seawater and brackish water using membrane technology is in high demand, mainly for invasive industries such as aquaculture and agriculture. Due to the limitation of water recovery of conventional membrane technology such as reverse osmosis (RO), they can be used to concentrate the salt ions and eventually generate a brine disposal problem. Furthermore, formation of polarization films and by-products may initiate bacteria growth and fouling in RO process [2,3]. A study investigated the extraction of Lithium-ion (Li^+^) found in the seawater using ion-selective membrane [4]. They discovered that the Li^+^ had successfully extracted with different movement depending on the nanochannel dimension, but for NaCl concentration, the permeate volume was only half of the permeate volume. The limitation of this conventional membrane may be overcome by using the alternative membrane technology such as membrane distillation.

Membrane distillation (MD) is a process that involves the separation of non-volatile compounds by generating vaporization and condensation processes [5]. In other words, the process inhibits the saline penetration and allows the water vapor passage [6,7]. Membrane requirements for MD process, i.e., high liquid entry pressure (LEP), high contact angle, rough surface, low thermal conductivity, thin and durable, along with the porous symmetric morphology play a crucial role in the selective transportation of salt ions and the efficiency of membrane performances [8]. Thus, it delays the intrusion of saline feed solution [9].

Wetting resistance is an important factor for the development of membrane for MD process to ensure an effective selectivity, water repellence and self-cleaning [10]. Several techniques such as phase separation [11], electrospinning [12] and surface modification [13] are currently adopted to fabricate an anti-wetting membrane. These techniques can be either fabricating an anti-wetting membrane or modifying the membrane into an anti-wetting membrane by low surface energy materials. Therefore, the hydrophobic polymers are introduced in membrane fabrication for the MD process, such as poly(tetrafluoroethylene) (PTFE), poly(vinylidene fluoride) (PVDF), polypropylene (PP), and polyethylene (PE), respectively. Among hydrophobic polymers, fluorinated polymers have been widely used due to the preference advantages. The fluorinated polymers have stable C−F bonds and strong C−C bonds, which relatively unreactive and chemically stable. The presence of high fluorine contents decreased the reactivity of these polymers [14,15]. However, using only fluorinated polymer in membrane fabrication has been reported to attain a few drawbacks in membrane wetting and fouling of hydrophobic and amphiphilic contaminants [16,17]. These drawbacks encourage the discovery of new materials that prevent problems and improve membrane performances.

Introduction of inorganic nanoparticle as filler in the fabricated membrane has been widely investigated to improve the anti-wetting properties using different methods [18,19]. However, a few has carried out by clay minerals. A previous study has investigated the fabrication of PVDF-Cloisite 15A for MD process, and obtained a hydrophobic contact angle with value of 97.72 ± 2.54° and color rejection of 95.33% [20]. The result may relate to the possible improvement of membrane selectivity by increasing surface anti-wetting. Besides, the clay minerals have a unique structure that consists of tetrahedral silica oxide and octahedral Al, Fe, or Mg oxide layers. Among clays, halloysite nanotube (HNT) is known as rolled kaolin with 1:1 phyllosilicate (alumosilicate group) and the rare nanotubes with a different composition of outer and inner surfaces. The outermost layer of the HNT consists of silica with a negative surface charge, whereas the inner lumen comprises of positive charge alumina [21,22]. The nanotube structure and less abundant surface hydroxyl groups facilitate the HNT particles to readily disperse in polymers without the exfoliation process, which contrasts with the platy clays (kaolin and montmorillonite) [23]. The presence of HNT provides an excellent orientation in the polymer matrix, which significantly improves the tensile strength, optical and mechanical anisotropy. The advantages of HNT in the polymer–clay membrane for the MD process requires a thorough study as its unique structure shows potential in removing salt and other contaminants.

Most of the applied fillers are hydrophilic materials, including HNT, which causes low anti-wetting properties to the MD membranes as presented by the Mokhtar and team study. In recent studies, researchers examined an application of fluoroalkylsilane (FAS) to prepare a high anti-wetting membrane for MD process, either by pre-treatment (on filler) or a post-treatment (on membrane) method [12,24]. However, surface wetting is still a significant ongoing issue. Researchers have tried to introduce natural wax to create anti-wetting surfaces with the formation of micro- or nano hierarchical structure for molecular sieving in aqueous solution [25,26]. Natural wax can offer water repellence to an extent that can reach one gained with fluorocarbons [27]. The availability of commercial carnauba wax and beeswax resulted in high usage in preparation of anti-wetting surfaces [28,29]. This carnauba wax contains esters (mainly fatty acids) and hydrocarbons. It is soluble in organic solvents and insoluble in water, which poses high durability in wear-resistant and waterproof properties (self-cleaning effect) [30]. It has the highest melting point compared with other natural waxes between 82 °C to 86 °C [31]. A recent study reported that coating with the presence of carnauba wax was more stable compared to the paraffin wax in terms of durability toward temperature [32]. The robust durability of carnauba wax may be generated with the combination of inorganic nanoparticles where it will associate and assemble into unique structures.

Hence, in this study, the carnauba wax was investigated with the common hydrophobic agent FOTS to determine the possible membrane characteristics for the MD process, specifically DCMD. The hydrophobic PVDF/f_s_-HNT (Pf_s_-HNT) and Pf_w_-HNT membranes were developed to observe the improvement of anti-wetting properties and membrane performances for the DCMD process. Besides, the study on carnauba wax as a hydrophobic agent is still limited. This study has encouraged the authors to investigate the effect of the f_w_-HNT particles in the PVDF matrix. The membrane characterization and performances, including permeability, solute rejection, and anti-fouling, were evaluated and discussed.

## 2. Materials and Methods

### 2.1. Materials

The hydrophobic membrane was prepared by poly(vinylidene fluoride) (PVDF) Solef 6010/1001 powder from Solvay Solexis (Aubervilliers, France). This PVDF powder was dissolved in triethyl phosphate (TEP, Merck, Darmstadt, Germany), which was used as a solvent. Halloysite nanotube (HNT) powder was functionalized by 1H,1H,2H,2H-perfluorooctyl-trichlorosilane (FOTS, 95%), and carnauba wax from Sigma Aldrich (Columbia, MO, USA). N-hexane (>99.9%) and methanol were acquired from Merck (Darmstadt, Germany). Distilled water was used as a coagulant bath during the immersion precipitation process and as feed solutions in DCMD operation with the additional solution of real aquaculture seawater near Penang bridge, 6 km away from the coastal area.

### 2.2. Functionalization of Halloysite Nanotube (HNT)

FOTS and carnauba wax were used according to the literature [33] with a few modifications. The 5 g of HNT was stirred in 12.5 g n-hexane solution consisting of 5 wt.% FOTS for 24 h. The functionalized HNT was then separated and dried in a vacuum oven at 100 °C for 1 h. The white powder of functionalized HNT should be dried in a vacuum oven at 60 °C to 70 °C for 24 h before further experiments.

For the carnauba wax functionalization process, the 5 wt.% wax was dissolved in 12.5 g n-hexane with continuous stirring at 85 °C until complete dissolution. A similar amount of HNT was then added to the wax solution and kept stirring for 24 h at room temperature. The drying process was carried out as in FOTS functionalization. This modified HNT changed its color to yellow powder due to the presence of carnauba wax.

### 2.3. Synthesis of Hydrophobic Membrane

TEP was used as a solvent in a polymer solution and heated at 70 °C for 15 min before adding 18 wt.% PVDF. The polymer solution was stirred and heated until the PVDF polymer became homogenous with the solvent. This polymer solution was left with continuous stirring and heating for 24 h. Then, the resulting polymer solution was degassed for 1 h to remove the air bubbles. This solution was cast onto a glass plate with a thickness of 500 µm. After the casting process, the cast polymer solution was immersed into pure methanol after 30 s exposure for 15 min in a cold surrounding (19 to 22 °C) and immediately transferred into a cold-water bath for 24 h. The solidified polymer solution was dried at room temperature and formed a membrane.

Similar steps were carried out for PVDF/HNT composite membranes with an additional method. The 5% of functionalized HNT relative to 18 wt.% of PVDF polymer was added in the one-third of 82 wt.% TEP and sonicated for 2 h at 40 °C. This HNT solution was then added to the dissolved PVDF solution with continuous stirring at 70 °C for 24 h and cast as the neat PVDF membrane.

### 2.4. Characterization of the Hydrophobic Membrane

The anti-wetting properties of the prepared membranes were repeatedly measured two to three times, particularly for the contact angle of the water (CA) and liquid entry pressure (LEP). The CA was evaluated by the sessile drop method using a goniometer (Rame-Hart 250 F-1, Succasunna, NJ, USA). Almost 6 µL water dropped on different areas of the membrane surface to measure the CA value. The readings were automatically recorded by the software.

The average LEP measurement was carried out by Porolux^TM^ 1000 porometer (Benelux Scientific, Nazareth, Belgium). Water is the most suitable liquid to measure LEP as it has higher surface tension, which inhibits the water molecules to absorb immediately into the pores. The LEP reading was taken after the first water penetration into the pores using nitrogen gas pressure.

Other than LEP, the porometer was also used to measure the mean pore size of the membrane. The membrane was placed in the porefil liquid for 1 h to ensure the pores were thoroughly wet and prepared the wetted membrane in the 20 cm sample holder. The calculated measurement from the air pressure and nitrogen gas flowing through the membrane pores were recorded.

The porosity of the prepared membrane (ε) was triplicated and calculated using Equation (1). The membrane of 1 cm × 1 cm was immersed in Porefil liquid for 1 h. The wet membrane was weighed (m_1_) and dried in the oven for 24 h. After the mass of the dry membrane (m_2_) was measured, the membrane porosity was calculated using all the information.

ε (%) = ((m_1_ − m_2_)/ρ_1_)/((m_1_ − m_2_/ρ_1_) + (m_2_/ρ_2_)) × 100% (1)

where ρ_1_ is the density of Porefil liquid (1.87 g/cm^3^) and ρ_2_ is the density of PVDF polymers (as the HNT content in the membrane matrix is small and ρ_2_ is approximate to ρ_PVDF_) (1.78 g/m^3^) [20,34].

The additional functional group from FOTS and carnauba wax molecules on HNT particles were analyzed using a Fourier transform infrared-attenuated total reflection (FTIR-ATR, Thermo Scientific Nicolet Nexus 670, Nicolet iS10, New York, NY, USA) measurement. The transmittance bands of PVDF membranes were recorded between wavenumber of 600–4000 cm^−1^.

The morphology of the prepared membrane was characterized via a scanning electron microscope (SEM, HITACHI S-3000N, Hitachi Ltd., Tokyo, Japan). Before analysis, the sample was immersed in liquid nitrogen to ensure that no pores were damaged during fracture and were sputter-coated with gold.

The roughness of the membrane was analyzed using atomic force microscopy (AFM, Park System XE100, Suwon, Korea). Surface roughness was measured by tip-scanning with a scan size of 20 µm × 20 µm. The XEI software was used to determine the mean roughness (R_a_) of the scanned area.

### 2.5. Membrane Performance via DCMD

**Figure 1:**
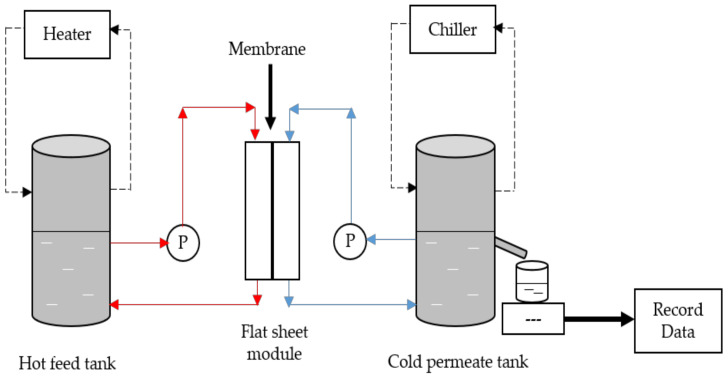
Set-up of direct contact membrane distillation (DCMD) operation process. The cross-flow membranes were carried out at constant temperature at feed and permeate sides for all the prepared membranes.

The experimental set-up was shown in Figure 1. The hot feed of 2 L distilled water was heated approximately at 60 °C, which pumped into the crossflow membrane module with an effective area of 47.5 cm^2^. The water flow rate was adjusted and controlled to 252.36 mL/min by a peristaltic pump. The water vapor that formed was passed through the membrane and condensed at the cold permeate side (20 °C) with a similar flowrate using a centrifugal pump. The prepared membrane was run twice for 16 h by a half-hour of distilled water and real aquaculture seawater with the conductivity of 36 to 40 mS/cm and at approximately pH 7. The water and permeate flux were calculated via Equation (2), while the percentage of salt removal equation had presented in Equation (3).

J = ∆W/(A·∆t)
(2)

where J is the flux permeation (L/m^2^h), ∆W is the permeate weight (L), A is the effective area (m^2^), and ∆t is the time in h.

R = (1 − (C_p_/C_f_)) × 100% (3)

where R is the percentage of salt removal. C_p_ is the permeate conductivity (µS/cm) and C_f_ is the feed conductivity (µS/cm).

## 3. Results and Discussions

### 3.1. Effect of Functionalized HNT on the Membrane Anti-Wetting Properties

Membrane thicknesses were cast at 500 µm gap height and obtained 217 µm for a neat PVDF membrane matrix after phase inversion process as shown in Table 1. Meanwhile, the PVDF/functionalized HNT membranes were found to be 245 µm and 285 µm for the Pf_w_-HNT and Pf_s_-HNT, respectively. Increase in thickness was probably due to the hydrophobic characteristic of functionalized HNTs, which delayed the demixing of solvent/non-solvent and resulted in a slow polymer separation phase. In contrast with hydrophilic filler [35], it could be speculated that hydrophobic functionalized HNT causes symmetric structure followed by a slow solvent/non-solvent exchange rate due to the hydrophobic characteristic of filler that prevent the fast leaching out of during the immersion precipitation process. The usage of methanol as non-solvent could be another reason for the increased thickness as it encouraged the formation symmetric membrane layer.

**Figure 2:**
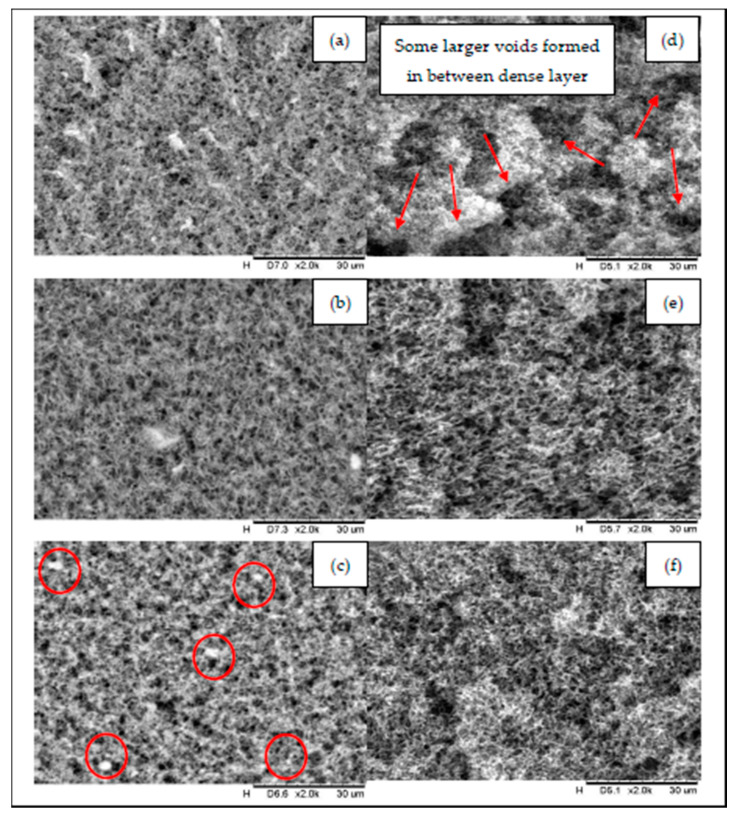
Comparison of surface (left) and cross section (right) of the prepared membranes before and after the addition of functionalized halloysite nanotube (HNTs); (**a**,**d**) PVDF, (**b**,**e**) Pf_s_-HNT and (**c**,**f**) Pf_w_-HNT.

Surfaces and cross-section morphologies of all prepared membranes (PVDF, Pf_s_-HNT and P_w_-HNT) are shown in Figure 2. In Figure 2, the neat PVDF membrane has a relatively denser membrane layer and rugged surface compared to Pf_s_-HNT and Pf_w_-HNT membranes, which could influence the membrane permeability. The formed morphology of the neat membrane might be due to the high polymer concentration that decreased the diffusion rate of the solvent/non-solvent and caused a compact layer with the lowest thickness. The formation of some larger voids between the inner layer of the neat membrane (refer Figure 2d) might influence the higher mean pore size values of this membrane. The addition of 5 wt.% of functionalized HNTs to the polymer solution results in a dense surface with narrow mean pore sizes (in a range of required pore sizes for MD). The f_s_-HNT particles had changed the density (Figure 2a) to a less dense interconnected thin fibrous-like membrane structure (Figure 2b). Meanwhile, the f_w_-HNT particles formed an interconnected thick fibrous-like membrane structure. A formation of well-distributed membrane pores on the membrane surface (Figure 2c) was also observed. These findings could be related to the nature of the applied polymer and the viscosity of the prepared polymer solution. The presence of hydrophobic functionalized HNTs increased the viscosity of the solution and decreased the exchange rate of the solvent/non-solvent in which the solvent and non-solvent slowly diffuse out of the PVDF/functionalized HNT solutions during phase inversion [36]. Therefore, the small pore size with applicable surface porosity of the Pf_w_-HNT membrane was formed as shown in Figure 2f. For cross-section morphology, the formation of sponge-like pores was ascribed to the increasing viscosity of the solutions. Increasing the concentration of the polymer solution could reduce the coagulation value due to the stronger interaction between the polymer and solvent and the slower non-solvent/polymer interaction. Previous study has explained the formation of the different cross-section morphology of 17.5 wt.% of polymer concentration with a different height-of-thickness gap [37]. From their findings, the viscosity of the polymer solution and thickness shows substantial influence toward the inner structure of the prepared membranes. They reported that the gap height lower than 600 µm could form the symmetric structure from the top to bottom layers due to the inadequate space for the macrovoid to grow longer and induce a porous substructure. Hence, in this study, the incorporation of f_s_-HNT into PVDF solution obtained the thickest membrane layer, which indicated the most viscous concentration of the polymer solution. It is suggested that due to the concentration of f_s_-HNT and PVDFm between 11–20% exhibit the particle–particle interaction between HNT and the polymer, which predominates a uniform distribution within the composite and form an interconnected network [38]. However, in the MD process, increase in membrane thickness may reduce the mass transfer coefficient as it requires a longer passage for the penetration of water vapor [39]. Thus, this membrane is not preferable compared to the Pf_w_-HNT membrane.

**Figure 3:**
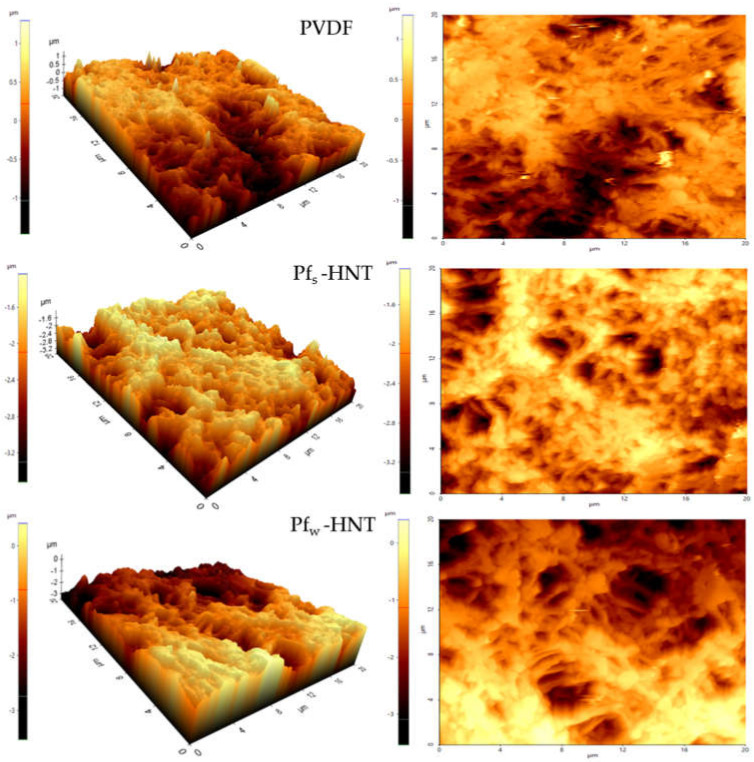
Three-dimensional and surface images of atomic force microscopy (AFM) for surface roughness analysis; PVDF, Pf_s_-HNT and Pf_w_-HNT membranes.

The surface roughness of the prepared membranes was carried out for the neat PVDF, Pf_s_-HNT and Pf_w_-HNT membranes as shown in Figure 3 using AFM analysis. The membrane roughness shows that the addition of f_w_-HNT has increased to two times higher than the neat membrane value. This event can be explained owing to the interaction between solvent and non-solvent, which formed the dense surface structures as shown in Figure 2c. The dense structure of entangled fibrous layers was observed to have white bead-like particles evenly attached on the fibrous-like structure (as marked by red circles). These particles might influence the improvement of surface roughness of this membrane compared to the Pf_s_-HNT membrane. A smooth interconnected fibrous-like layer and the less dense surface structure formed has lowered the surface roughness of Pf_s_-HNT membrane (Figure 2b) and led to a lower contact angle. Meanwhile, the agglomeration of the PVDF polymer on the membrane surface of the neat membrane might be the reason of the higher surface roughness compared with the Pf_s_-HNT membrane due to the high polymer concentration used, which could affect the membrane permeability. Thus, these SEM images agreed with the results obtained from the AFM analysis. Generally, the roughness of the membrane surface is correlated to the hydrophobicity of the membranes. An increase in surface roughness will increase the contact angle of the membrane surface, which defines the membrane hydrophobicity. The formation of a hierarchical structure may influence the high surface roughness. This structure will generate low surface energy between the liquid droplet and the solid surface and exhibit a weak adhesion force by promoting a high contact angle (>90°) [40,41]. This statement agrees with the obtained results for both PVDF and Pf_w_-HNT membranes, which exhibited an increased roughness with an increased contact angle of the membranes as shown in Table 1 and Table 2. However, the Pf_s_-HNT membrane was observed to reduce the surface roughness with R_q_ and R_a_ of 247 nm and 196 nm, respectively. The surface roughness of this membrane shows a contradict correlation with the obtained contact angle, which unusually occurred. In Figure 2b, the surface of the P_s_-HNT membrane was observed to have a smooth surface compared to the neat PVDF and Pf_w_-HNT membranes. The formation of a thin and smooth fibrous-like structure might influence the reduction in surface roughness of this membrane. However, due to the presence of f_s_-HNT in the polymer matrix (refer Figure 3 of surface image), it had improved the contact angle of this membrane. The result of the Pf_s_-HNT membrane could be related to the Wenzel state where the contact angle would increase and the contact line might be pinned at each wetted area as it receded, which is known as contact angle hysteresis. This consequence suggested that the presence of f_s_-HNT only formed a small scale of the surface roughness and insufficiently increased the contact angle as P_w_-HNT membrane. Thus, this membrane prompt to experience wetting phenomenon as it obtained the lowest surface roughness. In Figure 3, the three-dimensional and surface images of AFM analysis was obtained for the prepared membranes. AFM analysis of the PVDF membrane displays the formation of low aggregated on the surface of neat PVDF membrane. Typical nodular (hills and valleys) morphology of neat PVDF membrane was effectively intact even though the pure methanol concentration was used in a two-stage coagulation bath (methanol and water) [42]. These valleys were easily accumulated or blocked by contaminants or solutes during permeation and caused fouling. The surface image of the PVDF membrane observed a dense surface layer, which might block the pores and eventually disturbed the membrane permeability. For the Pf_w_-HNT membrane, the 3D images showed more lumpy aggregates compared to the neat PVDF membrane, which indicated the well-distribution of f_w_-HNT particles in the PVDF matrix and encouraged a more uniform membrane roughness. The increase in R_q_ and R_a_ from 396 nm and 325 nm to 765 nm and 635 nm indicated more formations of hills than valleys, resulting in a low possibility to trap contaminants. According to the surface image of the Pf_w_-HNT membrane, the interconnected fibrous-like layer was surrounded by a bead-like structure and did not overly cover the membrane pores. The denser surface morphology in Figure 2c might be due to the surrounded bead-like structure on the interconnected fibrous-like layer and thus increased the roughness of the membrane surface with high R_q_ and R_a_ values.

**Figure 4:**
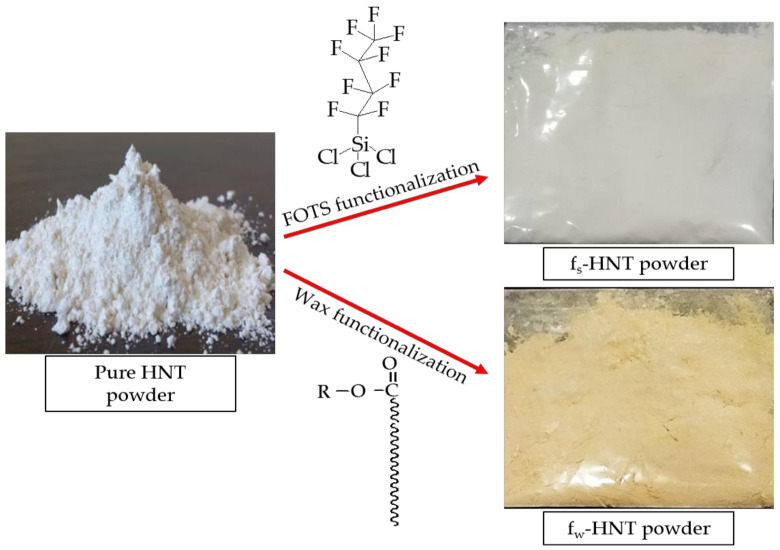
The change of powder color of pure HNT from white to yellowish after undergoing the functionalization process by carnauba wax (f_w_-HNT) and no color change was observed for 1H,1H,2H,2H-perfluorooctyl-trichlorosilane (FOTS) functionalization.

To further explore the effect of f_w_-HNT on membrane structure, the porosity and mean pore size of this membrane was measured. As shown in Table 1, the porosity and mean pore size of the Pf_w_-HNT membrane was lower than the PVDF membrane but obtained an insignificant difference to the Pf_s_-HNT membrane, which satisfied the SEM images shown in Figure 2. The results can be attributed to the following reasons. Firstly, in contrast to hydrophilic material [43], the hydrophobic material incorporated into PVDF matrix may slightly disturb the amorphous nature of the membrane, which generated strong points due to stresses and formed less fracture points that reduced the formation of pores. Additionally, the solid form of wax particles is another reason for the reduction of membrane porosity compared to the liquid form of FOTS, which might alter the formation of membrane pores as it also changed the original color of HNT powder from white to yellowish as shown in Figure 4. This possibly occurred due to the pore size decreasing as a consequence of the exchange interface between the solvent and non-solvent during membrane solidification process or the interaction among polymer chains [44]. Secondly, the solvent/non-solvent mass transfer rate was impeded due to the abundant hydrophobic groups on HNT, promoting the narrow pores formation. Finally, the introduction of f_w_-HNT changed the thermodynamic stability of polymer solution during solidification process and thus induced large liquid-liquid demixing gaps, resulting in the narrow pores [45]. These reasons have indirectly explained the possible occurrence that led to the outcome as in AFM images, particularly Pf_w_-HNT membrane.

**Figure 5:**
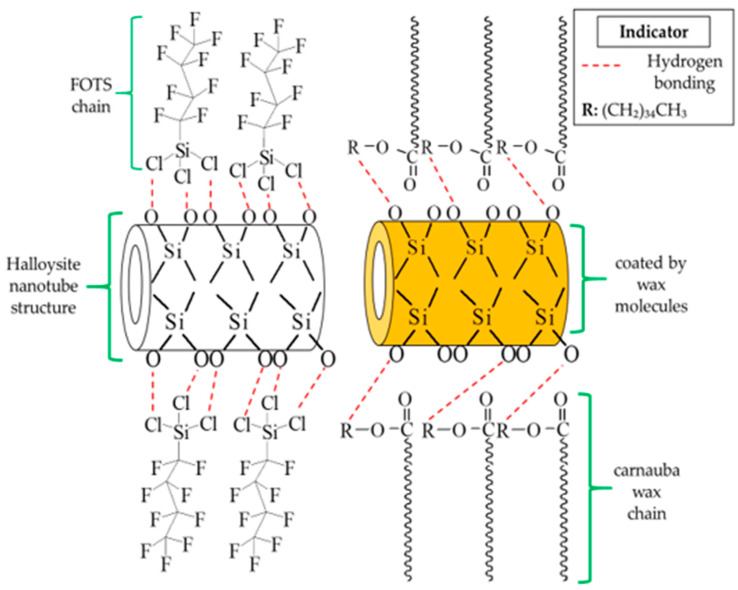
Illustration of proposed interaction of functionalized FOTS and carnauba wax with HNT. The hydrogen bonding takes places in both reactions of functionalized HNTs. The solid form of carnauba wax promotes the coating process on the surface of HNT particles. The functionalization process has facilitated the formation of hydrophobic HNT particles.

The reaction of functionalized FOTS and carnauba wax on HNT particles was proposed, as shown in Figure 5. The FOTS offered only a simple way to adsorb a compound by a hydrogen bonding reaction between fluorine chains and the SiO^−^ anion of the outermost layer of HNT particles [46]. Contrarily, the dissolved wax not only induced the exchange of fatty acid chains, but also covered the HNT particles. The FTIR analysis had proved the existence of fatty acid chains on the filler while the covering of f_w_-HNT compared to f_s_-HNT was observed to change the color powder from white to yellowish (Figure 4). Consequently, the covered wax on the HNT particles has caused a slight reduction in membrane porosity. In contrast with the polymer, the filler is likely to distribute in the polymer solution. The distribution of the filler may be influenced by the interfacial adhesion between the filler and polymer and the viscosity of the polymer solution. The good interfacial adhesion of f_w_-HNT with PVDF might induce a strong bond between both materials, which generates a small mean pore size of the membrane and indirectly reduces its porosity to 85%. The bonding attraction had slowed down the diffusion rate during the immersion precipitation since the viscosity increased with the presence of f_w_-HNT particles [47]. However, the previous study has reported the addition of HNT in poly(vinyl chloride) (PVC) and obtained only 52.92% for the highest membrane porosity [48], which is lower compared to this study. It can be explained because the incorporation of functionalized HNT by wax shows a good interfacial interaction with PVDF compared to the previous study and induces a better porosity of Pf_w_-HNT membrane up to 85%. Moreover, the negative surface charge of HNT had been efficiently coated by sequential adsorption of positively and negatively charged polyelectrolytes, thus, offering the diffusion barrier decelerating the release [49]. This previous study reported that the electrical charge had coated the polyelectrolytes, which prevents the HNT from leaching. Relatively, the negative surface charge could indicate the interaction between the SiO^−^ anion with the fatty acid chains of this functionalized HNT as in the proposed interaction that promoted the covering of wax. Thus, the good interfacial adhesion of this filler in the polymer matrix had prevented the leaching process of the filler in the non-solvent, which was attributed to the reduction of porosity from 87% to 85%. This was due to the additional wax that was significantly attached to the HNT particles (refer to Figure 4) and generated narrow pore sizes with increased membrane thickness (refer Table 1).

**Figure 6:**
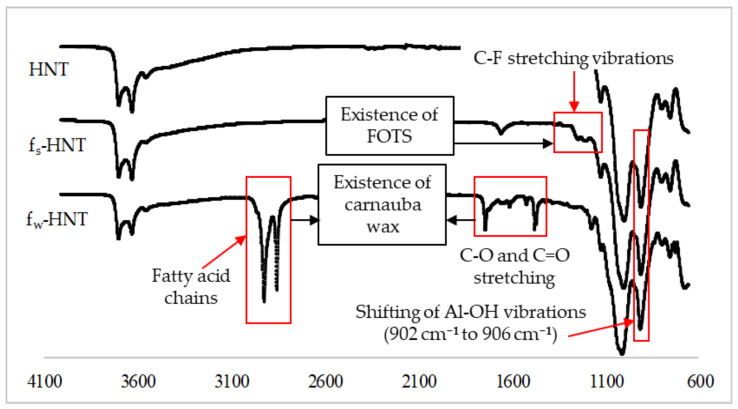
New functional groups of functionalized HNT particles using FOTS and carnauba wax. Carnauba wax functionalization shows a significant peak along the HNT spectra.

The proposed interaction was further justified by the FTIR-ATR analysis to confirm that the FOTS and carnauba wax chains were successfully attached to the HNT particles as shown in Figure 6. In general, HNT particles consist of Al-OH groups located at the ends of the nanotubes and on the inner lumens, while the outer layers were covered by Si-OH groups [23,50]. According to the FTIR spectra, the Al-OH groups of HNT particles were assigned around 902 cm^−1^. For the functionalized HNTs, the f_w_-HNT observed a slight shift of about 4 cm^−1^ for the band of Al-OH vibrations compared to an insignificant shift of f_s_-HNT as reported in the previous study analysis [51]. The hydrogen bonding could occur during the functionalization process due to the shift of the spectra absorption of Al-OH vibrations and Si-O stretching. The insignificant shift of the absorption of Al-OH vibrations might happen when there were only a few Al-OH groups at the end of the tubes [52]. The light new bands at 1240 cm^−1^ and 1180 cm^−1^ in f_s_-HNT spectra existed in the FTIR spectra, which indicated the C-F stretching vibration of FOTS chains bound to the surface of HNT particles [53]. For f_w_-HNT particles, the intense bands were observed at 2915 cm^−1^ and 2848 cm^−1^ for fatty acid chains. The C–O stretching vibrations of the functional group ester carbonyl and C=O stretching vibrations were present at 1734 cm^−1^ and 1471 cm^−1^, respectively [31,54]. Hence, all these bands significantly proved the strong interfacial reaction between carnauba wax and HNT particles compared to f_s_-HNT. The functional groups observed in the FTIR spectra were compatible with other results, SEM, AFM, porosity, and pore size, respectively.

The water contact angle (CA) was measured to evaluate the surface hydrophobicity of membranes that acted as an important role for the membrane permeability and anti-fouling behavior. The neat membrane had the lowest CA (112°), suggesting that it was less hydrophobic. As the functionalized HNTs were introduced, the CA increased to 127° and 137° (Table 1), implying a more hydrophobic surface of PVDF/functionalized HNT membranes due to the presence of fluorine and long carbon chains of f_s_-HNT and f_w_-HNT. During the membrane gelation process, the functionalized HNT might impulsively migrate to the membrane surface due to the light weight of HNT fillers and the high affinity of non-solvents compared to the PVDF polymer. The process promoted the formation of micropapillae on the membrane surface, which generated a rough skin layer and improved surface hydrophobicity. These micropapillae that were excited on the surface of water repellent nature had obeyed the Wenzel theory, which induced the CA value within 90° to 150° [55,56,57]. Additionally, the CA value was influenced by an uneven membrane surface, which was formed with a high content of hydrophobic HNTs. According to the functionalized filler characteristic, the HNT of wax-based particles showed a high tendency to agglomerate. The f_w_-HNT was observed to evenly agglomerated on the fibrous-like layer as seen in AFM surface image. From the AFM analysis, a high value of surface roughness (R_a_) of the Pf_w_-HNT membrane corresponded to the high CA value. The hydrophobic surface could reduce the membrane wettability due to the low surface energy and cause a weak adhesion force between the water droplet and the rough membrane surface [15]. Thus, low water liquid would penetrate the membrane surface.

For the MD membrane, other than CA, liquid entry pressure (LEP) is also one of the crucial factors of anti-wetting properties for an efficient MD process. These properties could delay the wetting phenomenon of the membrane. However, the surface roughness of the hydrophobic membrane could only influence the outer layer wettability, which is prone to cause the wetting phenomenon of an inner layer if the LEP was less than 1 bar. The results showed that the addition of the f_w_-HNT in the PVDF matrix had improved the CA of the membrane to 137° with an applicable range of LEP (2.69 bar) compared to the Pf_s_-HNT membrane, which was much lower. Even though the functionalized HNT increased the CA, it had caused a slight decrease in the LEP values as it involved the strength of the formation of inner geometrical structures. The high magnification of cross-section images proved the less dense layer by large voids formed in the inner structures of both Pf_s_-HNT and Pf_w_-HNT membranes compared to the PVDF membrane that led to the slight reduction of LEP. The LEP of the membrane was closely related to the pore size of the membrane. In this study, the intermediate value of LEP observed in the Pf_w_-HNT membrane could be correlated to the formation of a smaller pore size compared to the Pf_s_-HNT membrane, which indirectly facilitated the improvement of LEP. However, the highest LEP of the neat membrane was due to the absence of HNT particles and the large PVDF content in the membrane matrix. HNTs are more flexible than PVDF polymers and slightly reduced their endurance to some parameters due to the material’s limitation. Eventually, the drawback of this membrane might improve with the addition of 5–10 wt.% HNT content and providing double improvement of the composite strength [58]. Even though the neat membrane achieved the highest LEP, it showed some drawbacks in other evaluated properties, mainly in membrane permeability. Hence, the LEP value of the Pf_w_-HNT membrane observed an excellent potential for the wetting resistance of the MD membrane. Overall, the limitation studies in the fabrication of the PVDF/HNT membrane, specifically the Pf_w_-HNT membrane as an applicable membrane for the MD process, had shown the successful results for new hydrophobic membrane development on membrane characteristics that met the MD requirements.

### 3.2. Membrane Performance Using Real Aquaculture Seawater

**Figure 7:**
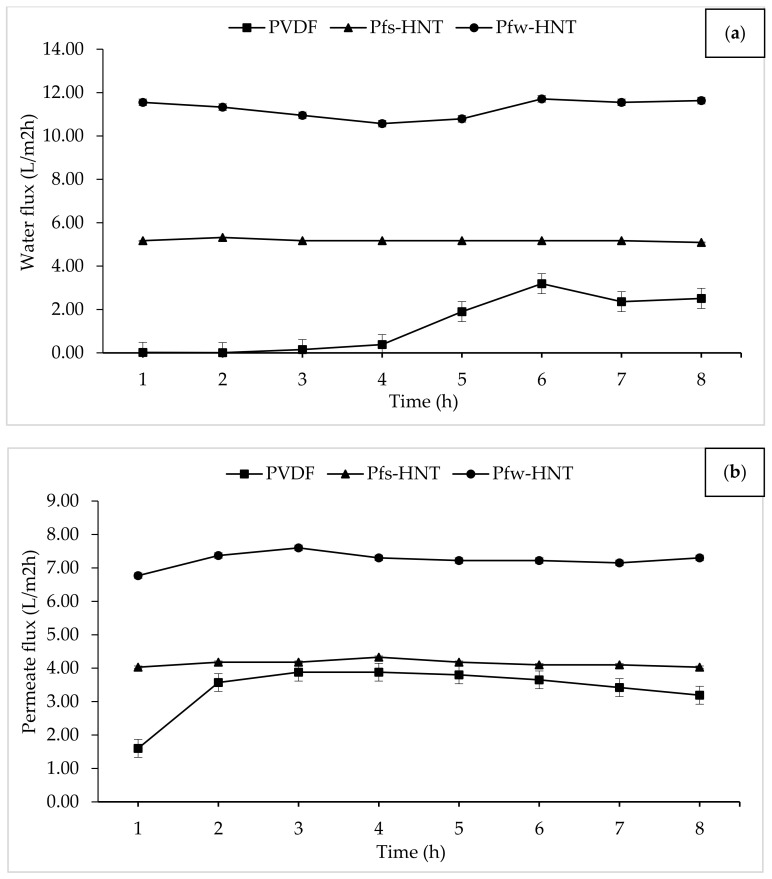
Evaluation of membrane permeability using water and aquaculture seawater. The prepared membranes were exposed to 16 h of MD process; (**a**) 8 h water vapor permeation by water and (**b**) 8 h of water vapor permeation from aquaculture seawater as feed.

DCMD is one of the basic MD configurations. The DCMD involves the vapor pressure difference across the membrane pores. This operation creates a vapor pressure difference across the membrane through different temperatures by vaporization and condensation processes [59]. Therefore, the application of hydrophobic PVDF/HNT membranes were carried out to determine the water vapor permeability of the aquaculture wastewater. In this study, the effect of f_s_-HNT and f_w_-HNT particles on the PVDF membrane was investigated. From Figure 7, a significant improvement in flux permeation of the fabricated membranes was observed after the addition of functionalized HNTs. The permeation was investigated under a constant temperature of 60 °C and 20 °C at hot and cold sides, respectively. For the neat PVDF membrane, the flux was lowest because of the dense sponge-like pores of the inner layer that caused some barriers and indirect flow to water vapor permeation. Even though the neat membrane obtained the highest porosity and pore sizes, the high density and viscosity of the pure PVDF solution created a denser inner structure with the least thickness layer. This compact layer could retard the flow of water vapor throughout the MD process. In contrast with the Pf_s_-HNT membrane, the presence of this filler increased the viscosity of the polymer solution that led to a high membrane thickness without altering the percentage of membrane porosity. However, the thickness of the membrane layer had promoted only a slight increase from the neat membrane. The Pf_w_-HNT membrane obtained a significant increase of permeate flux for both feeds (water and real aquaculture seawater). The increased flux of the Pf_w_-HNT membrane compared with the PVDF and Pf_s_-HNT membranes was attributed to the improved hydrophobicity and enhanced interconnected fibrous-like structure. It could be implied that the alteration of the membrane structure was the vital factor for the membrane performance [60]. The dispersion of functionalized HNT particles in polymer solution is the main factor for the microstructure formation and membrane performance. When the content of functionalized HNT particles exceed the applicable amount, the coagulation of the particles slowed down and resulted in an increased membrane thickness (Pf_s_-HNT membrane) with a declination of membrane permeability.

**Figure 8:**
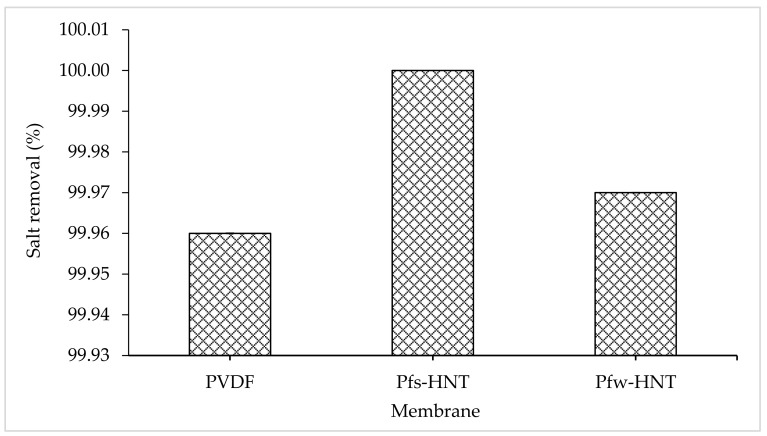
Percentage of salt removal of the prepared membranes after 8 h of DCMD process. All the prepared membrane achieved >99% of salt removal throughout the continuous operation.

From the flux permeation graphs, the average flux of both water and aquaculture permeates had tabulated in Table 3. The average water flux obtained an ascending order of 1.32 L/m^2^h to 5.18 L/m^2^h and 11.26 L/m^2^h for the neat PVDF, Pf_s_-HNT and Pf_w_-HNT membranes, respectively. Meanwhile, the permeate flux showed some decrement to 4.14 L/m^2^h and 7.24 L/m^2^h for both functionalized HNT membranes, Pf_s_-HNT and Pf_w_-HNT, except for the neat membrane. The neat PVDF membrane observed an unstable permeability for a long continuous MD process (16 h), which was further identified by the standard deviation measurement. The resultant flux permeation was suggested due to the pore swelling phenomenon that increased the vapor permeation after 8 h of water feed [61]. This phenomenon could be related to the adsorption of ions from the salt solution onto the membrane surface with a consistence to the AFM analysis on the formation of valley morphology. The increasing salt concentration could increase the membrane charge density, which led to an increase or decrease of surface charge density depending on the adsorbed ions. Thus, a higher or lower concentration of counterions inside pores occurred to neutralize the surface charge, as this effect induced the electrostatic repulsions between counterions [62]. However, the addition of functionalized fillers had highly facilitated the flux stability with the standard deviation <0.5. The Pf_s_-HNT membrane obtained the most stable flux for both feeds with 5.18 L/m^2^h ± 0.06 and 4.14 L/m^2^h ± 0.10, respectively. Even though the Pf_w_-HNT membrane was less stable in flux permeation than the Pf_s_-HNT membrane, it is still applicable to use as the MD membrane as it achieved up to 11.25 L/m^2^h (water) and 7.24 L/m^2^h (permeate) with an insignificant difference in salt removal (>99%) as shown in Figure 8. The effect of functionalized HNTs has inhibited the pore swelling phenomenon, even the membranes exposed to the high salt concentration solution. Abdel-Karim et al. reported that the flux of PVDF MD membrane using reduced graphene oxide (rGO) had improved the flux by approximately 7.0 L/m^2^h [63]. It convinced the flux permeation of this study, which was applicable for the MD process with further improvement.

**Figure 9:**
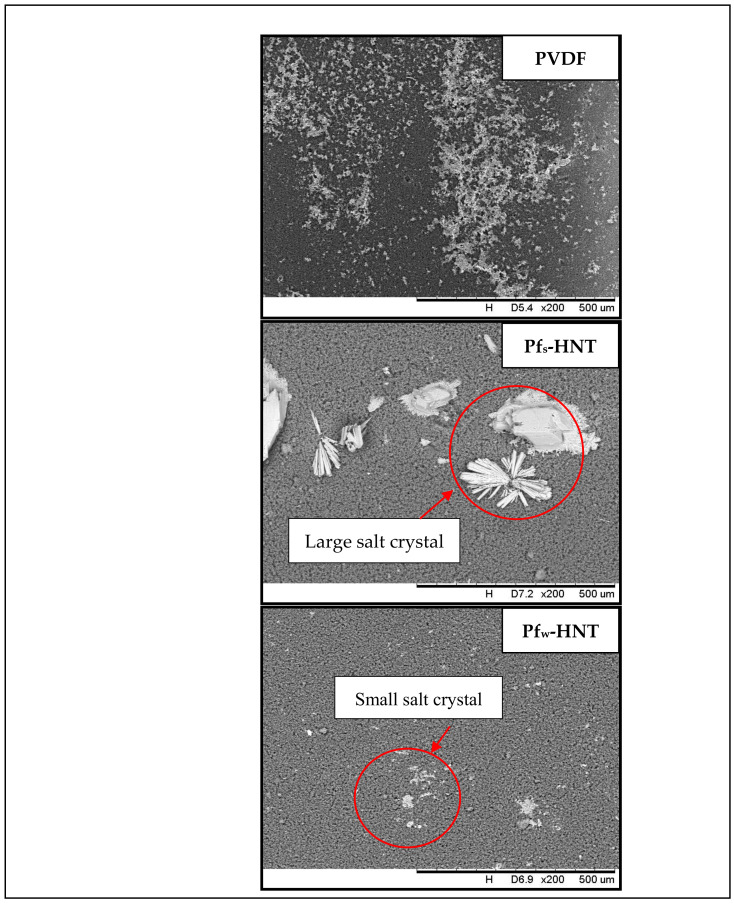
The reduction of salt scaling after introduction of functionalized HNT particles. Severe fouling on the surface of neat PVDF membrane was shown after 8 h of DCMD process. Addition of different functionalized HNTs had caused some adsorption of different size of salt crystals which indicated an improvement in anti-fouling behavior.

Even though the neat membrane obtained 99.96% of salt removal, the pore swelling phenomenon had led to the surface fouling due to the high adsorption of ions onto the membrane surface, as shown in Figure 9. The salt ions almost covered all the surface area of the membrane with the presence of white particles. This event might decrease the permeate flux with prolonged hours. Consequently, the pores wetting phenomenon might occur and reduced the membrane selectivity. In contrast with functionalized HNT membranes, the membrane surface of both membranes showed an anti-fouling behavior. The crystallization of large salt crystals was formed on the Pf_s_-HNT membrane and a few small crystals on the Pf_w_-HNT membrane. The mild salt scaling on both membranes could initiate an extended hour for the wetting phenomenon to occur compared to the neat membrane. The result showed that the Pf_w_-HNT membrane obtained an advantage property as an appropriate membrane for MD process.

Therefore, the introduction of carnauba wax as the hydrophobized agent for the HNT particles showed a high potential to tackle the fouling issue compared to PVDF and Pf_s_-HNT membranes. The Pf_w_-HNT membrane had identified achieving better anti-wetting properties and membrane performances. Wax has the potential to provide an effective barrier in limiting the diffusion of water liquid and aggressive ions as it could form an air layer at the interface and the low surface energy behavior [64,65]. In other words, the wax functionalization on HNT particles limits the salt ion diffusion, which indirectly encourages the water vapor permeation to pass through the membrane pores at a fast rate. Hence, there is increased water vapor permeation and improved anti-fouling behavior. Besides, the wax chains in the membrane matrix improved the membrane permeability and withstands a higher liquid pressure than FOTS chains. This result described the possibility of the Pf_w_-HNT membrane to have better shelf-life compared to the Pf_s_-HNT membrane. The Pf_s_-HNT membrane might be considered for future improvement as it achieved satisfied membrane characteristics and performance.

## 4. Conclusions

Hydrophobic membranes have been applied in various applications, particularly the MD process. Regardless of the MD requirement, the applicable MD membrane that meets the process requirements are still lacking to implement for industrial purposes. Therefore, the f_w_-HNT particles have provided desired anti-wetting properties, good anti-fouling behavior, and meet the performance for MD. The Pf_w_-HNT membrane has achieved the highest CA (137°) among the other fabricated membranes. Even though it has a lower LEP than the neat membrane, the 2.69 bar of LEP value has achieved the range of applicable properties for the MD and is higher than the Pfs-HNT membrane. The permeability of Pf_w_-HNT has improved up to 11.26 L/m^2^h and 7.24 L/m^2^h for water and aquaculture seawater permeates, with 99.97% salt removal. The modification by wax functionalization has developed a self-cleaning property after 16 h of the continuous MD process. Hence, this work offers new development of anti-wetting and anti-fouling membranes with optimum stability, which might be used further in long-term operation process.

The development of this membrane is aimed to be applied for wastewater treatments, specifically in aquaculture industry either inland or in seawater. The inland aquaculture wastewater is classified as brackish water, which also contains salt molecules. As both areas are comprised of salt molecules, this membrane was evaluated to acknowledge the potential of salt removal. The hydrophobic membrane for the MD process is not limited to salt removal, but it can be further investigated on other contaminants that exist in the aquaculture wastewater. Therefore, this Pf_w_-HNT membrane should be further improved in future works. There are some suggestions that can be studied for improvement, such as the introduction of hydrophilic additives, coagulation bath ratio and surface modification. These possible future works will promote a better development of superhydrophobic membranes in term of permeability, selectivity and wetting resistance, which may have the tendency to be applied in the real aquaculture industry.

From economic point of view, the implementation of low cost nanoclay and natural wax show cost-saving opportunities. These materials may not only reduce the cost of inventory raw materials but indirectly decrease the utilities cost of the production management. The common metal oxide nanoparticles are very expensive compared to nanoclay particles. The materials cost may reduce almost 10 times lower when nanoclay is applied. A similar consequence is attributed to the hydrophobized agents, comparably priced natural wax and fluoroalkylsilane. The natural-based material is not only cost-effective but it also gives low risks of an environmental impact, which obliquely cuts the potential risk cost. Other than raw materials, the introduction of the DCMD process is also one of the alternatives to decrease the cost of operation treatment, as this process not involve any external applied pressure. Recently, the electricity cost has been reduced by the introduction of solar system in the operation process. Hence, this study can be attributed as a good potential development in achieving the economical expectation of new invention and production in the future.

## Figures and Tables

**Table 1:** Anti-wetting and Pore Properties of the Prepared Membranes.

Membrane	Contact Angle (°)	LEP (bar)	Mean Pore Size (µm)	Porosity (%)	Thickness (µm)
PVDF	112 ± 0.55	3.10 ± 0.02	0.21 ± 0.05	87 ± 0.87	217 ± 0.01
Pf_s_-HNT	127 ± 1.07	1.51 ± 0.07	0.16 ± 0.10	87 ± 0.23	285 ± 0.01
Pf_w_-HNT	137 ± 0.91	2.69 ± 0.15	0.14 ± 0.07	85 ± 1.64	245 ± 0.01

**Table 2:** Comparison of the surface roughness between the neat PVDF, Pf_s_-HNT Pf_w_-HNT membranes.

Membrane	Surface Roughness Parameter (20 µm × 20 µm)	
	R_q_ (nm)	R_a_ (nm)
PVDF	396	325
Pf_s_-HNT	247	196
Pf_w_-HNT	765	635

**Table 3:** Average flux of water and aquaculture seawater permeate.

Membrane	Average Water Flux (L/m^2^h)	Average Permeates Flux (L/m^2^h)
PVDF	1.32 ± 1.31	3.37 ± 0.76
Pf_s_-HNT	5.18 ± 0.06	4.14 ± 0.06
Pf_w_-HNT	11.26 ± 0.43	7.24 ± 0.23
